# Supplementary material for: Complexome profiling of the Chlamydomonas psb28 mutant reveals TEF5 as an early PSII assembly factor
Source: Plant Cell. 2025 Mar 17;37(6):koaf055. doi: 10.1093/plcell/koaf055 (PMC12164590; doi:10.1093/plcell/koaf055)
Supplement: koaf055_Supplementary_Data [file koaf055_supplementary_data.zip › psb28_interactive.zip!/plots/intensity/qualityPlots/peptides/METE_Cre03.g180750.t1.2.html]

### Cre03.g180750.t1.2

Trivial name: METE  
SeqCoverage [median]: 8.30  
Mass: 86.77 kDa  
  
 

| Sequence | MissedCleavage | Mass [Da] | wtIntensity | muIntensity | Score |
| --- | --- | --- | --- | --- | --- |
| AAMLSTTTIGFPR | 0 | 1364.71 | 1698.32 | 0.00 | 88.68 |
| ALLDTGAVSSDR | 0 | 1203.61 | 336.77 | 1356.93 | 125.75 |
| GAALGAAAGTSR | 0 | 1001.53 | 613.11 | 559.82 | 116.30 |
| LAASPAAAAAAAGHGAVVQLQR | 0 | 2000.09 | 0.00 | 2380.79 | 45.09 |
| LGAGVVDGR | 0 | 842.46 | 1883.91 | 4885.71 | 130.27 |
|  |  |  | 4532.1 | 9183.2 |  |
